# Supplementary material for: Are carbon emissions trading and green financial instruments synergistic? -Comprehensive quantitative research based on content analysis
Source: PLoS One. 2024 Mar 7;19(3):e0298601. doi: 10.1371/journal.pone.0298601 (PMC10919601; doi:10.1371/journal.pone.0298601)
Supplement: S1 Data — (ZIP) [file pone.0298601.s001.zip › Data/地级市Z_F工作报告.zip!/┴╔─■/╕╖╨┬╩╨/└┤╫╘╣1⁄2╓┌║┼í╛┬φ┐╦╩2╛▌═°í┐_2005.docx]

各位代表：

　　现在，我代表市人民政府，向大会作政府工作报告，请予审议，并请市政协各位委员和其他列席同志提出意见。

　　一、2005年及“十五”政府工作回顾

　　过去的一年，在市委的正确领导下，全市上下高举邓小平理论和“三个代表”重要思想伟大旗帜，紧紧围绕推动超常发展和构建和谐阜新两大主题，抢抓机遇，开拓进取，扎实工作，经受住一系列重大事件的严峻考验，保持了经济社会全面发展的良好局面，较好地完成了市十三届人大三次会议确定的各项任务。

　　预计全市生产总值完成150.6亿元，增长16%，其中，一、二、三产业分别增长16.7%、14.5%和17.1%；全社会固定资产投资完成73亿元，增长25.2%；财政一般预算收入实现7.4亿元，可比口径增长16.5%；社会消费品零售总额66亿元，增长15%；城镇居民人均可支配收入6630元，扣除物价因素，实际增长16.2%；农民人均纯收入3140元，增长20.3%。

　　以2005年为标志，我们较好地完成了“十五”各项任务和目标。可以说，进入新世纪的五年，是阜新经受住严峻考验、在困境中崛起、实现历史性转折的五年。全市人民励精图治，奋力拼搏，赢得并紧紧抓住资源型城市经济转型试点市重大发展机遇，经济社会发展取得显著成就。主要体现在：经济转型取得重大阶段性成果，“主导产业+支柱产业”经济格局框架初步形成；全市生产总值结束了“九五”以来低速徘徊局面，年均增长17%，提前一年实现“十五”经济总量翻一番目标；地方财政一般预算收入年均增长17.7%（可比口径）；全社会固定资产投资累计完成249.5亿元，是“九五”的2.2倍；累计直接利用外资1.15亿美元，是“九五”的4.3倍；城镇居民人均可支配收入和农民人均纯收入年均分别增长10%和24.3%；累计实现就业19.5万人；社会保障体系不断完善；城乡面貌发生重大改观；生态环境得到明显改善，累计植树造林321.5万亩，是“九五”的3.9倍。这些成就，标志着阜新在转型复兴的伟大征程上迈出了坚实的一步，使全市人民对阜新的未来充满信心！

　　回顾过去的一年，主要抓了以下十一个方面工作。

　　（一）实施项目牵动战略，加快构筑“主导产业+支柱产业”经济格局。进一步加大力度，突出抓好项目建设，全市投资1000万元以上在建项目203个，总投资194.5亿元，其中，新开工111个，当年竣工59个。一是农产品加工业龙头企业不断壮大。阜新双汇销售收入达到12亿元，成为东北地区最大的肉类加工企业；阜新大江、阜新伊利、美中鹅业等一批企业销售收入突破亿元；华禾鹅业、关东肉羊、金隆木业、民安肉羊等14个项目开工建设；辉山乳业二期、铃兰味精一期、飞雪木业二期等11个项目竣工。二是推进了一批煤炭、电力工业项目。阜新发电厂三期改造、金山热电等一批投资20亿元以上项目进展顺利，特别是电厂三期在东北地区第一个实现当年主体工程暖封闭，创东北地区电力工程建设最好水平；金山热电完成投资进度的34.1%；百万千瓦风力发电项目规划初步完成，与华能公司签订了建设50万千瓦以上风电场合作协议。阜矿集团开发内蒙古白音华煤田项目获国家发改委正式批复，投资16亿元的阜矿集团60万吨甲醇项目通过省发改委立项。投资50多亿元的巴新铁路前期工作取得实质性进展。三是推进了一批优势产业项目。全市实施技改项目102项，技改投资增长85.6%，争取省技改贴息突破5000万元。鹰山水泥日产4000吨新型干法熟料项目一期、大金集团火电锅炉主体悬挂支撑系统、太克液压公司年产15万台叶片泵等15个新开工项目竣工投产，广厦钢构三期、德尔公司40万台汽车动力转向泵等52个项目开工建设。国家确定的23个转型重点项目有1项通过国家验收，13项达到国家验收标准。资源开发补偿机制和衰退产业援助机制试点相关政策争取工作正在积极推进。

　　（二）大力推进农业结构战略性调整，农业生产创历史最好水平。围绕农民增收，加快发展畜牧业、特色种植业、林果业和设施农业。粮食总产量187万吨，增长18.4%。畜牧业保持快速发展势头，畜牧业产值占农业产值比重达到44.9%。按照分类推进原则，加大瘦肉型猪、奶牛等14个农产品基地建设。猪、牛、羊、禽饲养量分别达到335万头、25万头、155万只和3000万只。奶牛饲养量突破3万头，白鹅饲养量达到840万只。杂粮、南瓜、食用菌、花卉、药材等特色种植业稳步发展，占总播种面积的25%。新发展蔬菜保护地4000亩。新植牧草7.6万亩。新植果树2.4万亩，水果产量13.4万吨。以户均一个致富项目为载体，启动百万农民增收致富工程，全市从事致富工程项目户数达到13万户，新增6万户。五大示范工程继续推进，落实无公害农产品基地364万亩，完成有机食品认证7个，实现零的突破；林下经济开发92万亩；新建“四位一体”生态模式户5050户。四大体系建设不断完善，市动物卫生监测检验中心交付使用，曾祖代猪场步入全国重点种猪育种行列。植树造林75.6万亩。封山禁牧工作取得阶段性成果，全年性和季节性封禁面积达到450万亩。完成以东北黑土区综合防治试点为主的水土流失治理面积25万亩。完成农村防氟改水工程90处。机械化保护性耕作面积78.3万亩，继续保持全省领先地位。实施人工增雨5亿立方米。农业综合开发工作在推动农业产业化、带动农民增收等方面发挥了重要作用。农村税费改革成果进一步巩固，农民负担没有出现反弹。落实惠农政策，发放粮食直补和良种补贴及农机具直补5857万元，增长48.4%。农村信用社改革完成阶段性任务。

　　（三）推进国企改革，工业经济实现持续快速增长。以产权制度改革为核心，通过挂靠联合、产权招商、股份制改造等形式，完成各类国有企业转制38户，处理壳企业17户。地方正常生产经营的一般竞争性国有工业企业基本退出国有序列。非工业系统国企转制工作全面启动，商业系统15户国有企业完成转制。市电缆厂、市氟化学总厂等7户企业列入国家正式破产计划，市矿山机械厂、市化工厂破产工作启动。海州露天矿关闭破产工作平稳推进，社会职能移交工作全部完成，伤残鉴定、退休审批、发放安置费等职工安置工作基本完成；通过阜矿集团内部挖潜，市劳动和工、青、妇等部门提供就业岗位，增加社区公益岗位等三个渠道，2300多人实现了再就业；6072户特困集体职工家庭纳入低保。加大工业经济运行监控，全市规模以上工业总产值增长25%，其中，地方工业增长38.3%。规模以上工业企业223户，新增24户；销售收入超亿元企业25户，新增6户。玻璃、橡胶、氟化工、电子工业园区基础设施和项目建设取得较大进展。阜矿集团采取组织矿井技术改造等一系列措施，生产经营稳步上升，为全市社会稳定、经济发展作出了重要贡献。

　　（四）以招商引资为重点，对外开放取得新成效。以项目为核心，突出重点国家和地区，突出重点产业，加大招商引资力度。全市招商引资到位38.1亿元，增长23%，其中，直接利用外资3850万美元（其中，新口径1328万美元），增长49%。先后组织和参加赴新加坡、马来西亚、美国、加拿大、西班牙、台湾、上海、温州、乌鲁木齐、济南等50多次境内外招商活动，取得较好效果。利用外资的规模和质量进一步提高，到位超100万美元的项目12个，比去年增加2个，其中工业项目7个。美国隆迪、飞雪木业、北方力劲等一批利用外资项目竣工投产，新天地商业广场等一批利用外资100万美元以上项目开工建设。积极应对国际市场粮食价格变动影响，调整出口产品结构，木制品、纺织服饰产品、毛革制品、农产品深加工等产品出口规模扩大，全年出口创汇3300万美元，超额完成省定指标。对外经济合作取得较大进展，全年境外输出劳务增长21%；积极争取国际无偿援助，彰武县第二人民医院50万元国际无偿援助资金全部到位。阜新经济技术开发区加大招商引资和服务体系建设力度，连续两年被评为全省发展较快开发区之一，新引进投资1000万元以上项目12个。阜新国家农业科技园区申报省高新技术产业开发区顺利通过省专家评审，一批农产品加工和机械加工项目进驻园区。

　　（五）县区经济发展速度加快，民营经济迈出新步伐。制定出台《关于支持县区经济发展，规范财税秩序的意见》，进一步加大支持力度，创造发展环境，有力地促进了县区经济的发展。县区生产总值平均增长24.3%，总量占全市比重达到52.4%，比上年提高2.4个百分点；农业科技园区、新邱区、海州区、细河区生产总值增幅超过30%；农业科技园区、细河区、彰武县、新邱区财政收入增幅超过25%。鼓励和引导民营经济参与国企改革，民营经济发展步伐加快，增加值、实缴税金分别增长25.8%和25.9%；新增销售收入超5000万元企业6户，其中超亿元企业2户

　　（六）积极推进各类市场建设，服务业发展迈出新步伐。辽西最大的家具和装饰材料市场昊海家居城投入使用，新建和改建一批农贸专业市场。阜彰两县全国“万村千乡”市场工程试点县建设走在全国前列，新合作·客多来配送中心、连锁超市等达到81家。加大了旅游工作力度，海棠山晋升为国家级自然保护区，海州矿矿山公园被列为首批国家矿山公园项目，十家子玛瑙市场被评为全国工业旅游示范点。全地区旅游总收入和外汇收入分别增长19.1%和18.1%。房地产开发业稳步发展，开工建设住宅面积122万平方米，房产交易面积137.9万平方米。信息化工作进入全省前列，软件业收入达到1.3亿元，增长33%。社区服务、中介服务、邮政、通讯等行业有了新的发展。

　　（七）认真做好财税金融工作，对地方经济发展保障能力进一步增强。加强财源建设，加大对重点企业、重点项目的支持力度，积极涵养税源。加强和规范财税管理，积极组织各项收入，严厉打击偷、漏、欠税行为。财政部门积极筹措资金，保障了全市重点支出的需要。国税收入增长22.1%，地税收入增长18.2%，均创历史最好水平。审计部门在强化资金监管等方面发挥了积极作用。全市金融机构新增贷款22亿元，增长35%，中行、商行、建行等在支持重点项目建设中作出较大贡献。电业、烟草、石油等中省直部门和单位在支持地方经济发展中发挥了重要作用。工商、质量技术监督等部门在加强市场监管等方面贡献了力量。

　　（八）加快城乡基础设施建设，人居环境和城市功能明显提升。我市第一座立交桥——阿金公铁立交桥主体工程基本完工。阜新旅客列车新客站主体工程建成。沈阳至彰武、阜新至铁岭高速公路开工建设，阜新至朝阳高速公路征地动迁核量工作完成。对东风路、创业路等4条城市主干道和8条街巷路进行了改造，建设道路桥梁23座。为550栋旧楼铺设连街甬道工程全部结束。细河5号坝主体工程完工。推进地下管网改造工程，改造供水管网25.3公里，新增供热面积30万平方米。污水处理工程基本达到试运行条件。引白水源项目部分工程开工。完成通村油路工程109公里。全国面积最大、投资最大的海州矿排土场土地复垦项目基本竣工，形成598万平方米绿地。海州矿地质灾害治理纳入国家计划并启动实施。城市植树30.3万株，新增城市绿化面积85.4万平方米，城市绿化覆盖率达到38.6%，人均公共绿地面积达到7.8平方米。深入推进“城市管理年“活动，制定出台了住宅小区建设与物业管理、城市噪音整治等一系列制度措施，清理和整顿了全市129条道路的秩序和环境，城市环境综合整治定量考核在全省排名上升1位。城市空气环境质量有所改善，全年65%以上的天数达到二级标准。小城镇建设取得新进展。

　　（九）关心广大群众切身利益，加快构建和谐阜新步伐。实施“五大民心工程”，结合开展保持共产党员先进性教育活动，在年初确定[Page]20项为民办实事的基础上，增加为民办实事14项，为弱势群体解决难题19项。启动实施棚户区改造工程，拆除棚户区面积121.5万平方米，开工建设205栋、79.7万平方米住宅楼，竣工回迁安置面积44.3万平方米，8300户居民迁入新居（含货币化安置）。采煤沉陷区治理工程接近尾声，当年竣工面积19.8万平方米，2601户沉陷区居民迁入新居；全市累计15141户沉陷区居民迁入新居，完成总回迁量的82.6%。全市实现就业和再就业5万人，其中，“零就业家庭”就业2.7万人；城乡劳务输出17.3万人次，其中输出城镇下岗职工1.3万人。人力资源大厦竣工。以扩面工作为重点，推进了社保体系建设。基本养老保险人数达到21.9万人，失业保险参保人数达到22.5万人，医疗保险人数达到30万人。启动实施了农村最低生活保障制度、农村低保大病救助制度、城市低保大病救助制度、城市医疗救助制度试点、城市低保分类救助制度等5项救助制度，有5.9万名群众受益。市慈善总会成立。住房公积金在解决中低收入群体住房方面发挥了重要作用。第六届社区居委会换届选举工作顺利完成。开展了“社区文化年”活动，社区办公用房基本达标，建设和完善42个社区卫生服务站并投入使用。完成农村中小学危险校舍改造15.1万平方米，资助义务教育阶段城市困难低保户和农村贫困户家庭子女就学7万人次。对56个重点村实施扶贫开发。

　　（十）大力发展科技教育，各项社会事业取得新成效。加强与高等院校、科研院所合作，举办了阜新市与沈阳工业大学、沈阳农业大学等10所大学科技项目对接洽谈会，继续推进与省农科院科技共建活动，辽工大为阜新转型提供了智力支持。引进推广农业新品种108个、新技术25项。与山东寿光农业专家协会共同开发的“阜新市农业科技服务系统”开通运行。农业科技成果转化中心交付使用。全面推进中小学教育信息化工程建设，城市中小学和农村中心校全部实现“校校通”。调整农村中小学24所，新建九年寄宿制学校10所。完成了市蒙中、二十一中学、海州高中校舍改造工程。市实验中学被评为2005年中国百强中学。“一村一名大学生”人才培养工程第二个五年计划正式实施。现代京剧《血胆玛瑙》代表辽宁省进京参加纪念抗日战争暨世界反法西斯战争胜利60周年全国优秀剧目展演，获得圆满成功；群众性文化活动蓬勃开展，建成两个省级乡镇文化中心和10个标准文化广场。完成农村“有线电视村村通”工程6万户，实现广播电视安全优质播出。篮球学校运动员公寓交付使用。市紧急救援中心和市传染病医院异地新建工程投入使用。阜蒙县新型农村合作医疗试点正式启动。农村部分计划生育家庭奖励扶助制度试点工作受到省委、省政府肯定。加强社会治安综合治理，全面启动社会治安巡逻防控体系。认真落实安全生产责任制，强化基础管理和专项整治，安全生产形势明显好转。工会、共青团、妇联等群团组织在推进经济社会发展中发挥了重要作用。进一步加强了统计工作，妇女、儿童、老龄、残疾人事业和物价、档案、消防、地方志、气象预测预报、地震监测等工作取得新成效。

　　（十一）加强民主法制建设，政府自身建设取得明显成效。自觉接受人大的法律监督和政协的民主监督，及时办理人大代表议案、建议和政协提案，办结率100%，满意率90%以上。从制度建设入手，努力建设依法行政、高效务实、亲民诚信、阳光透明、清正廉洁的政府。制定出台了32项有针对性、有约束力的制度措施，有力地推动了政府工作。继续深化行政审批制度改革，对全市169家收费单位的收费项目和标准进行了清理，对20项收费项目进行了减免。市行政服务中心全面实行限时办理和限时服务制、行政审批全程代理制，40个部门、312项行政许可及非行政许可审批项目入厅办理。在全省率先成立了市企业投诉中心，受理投诉案件23件，全部办结。设立市长信访接待日和接听公开电话日制度，8位副市长共接待群众来访40次，涉及78个问题，有69个问题得到解决；8位副市长共接听市长公开电话245件次，已办结211件，办复34件。《行风热线》栏目开办113期，接听群众咨询和投诉946人次，办结率98%。建立政府信息公开制度，通过市场化运作在街心广场设立政府信息公开电子大屏幕并投入使用；全年举办新闻发布会10次；建立和完善15条全方位听取意见的渠道，听取和采纳意见420多条。扎实开展了治理教育乱收费、向企业乱收费、公路“三乱”，纠正医药购销中的不正之风、巩固减轻农民负担成果等涉及群众切身利益的纠风专项治理，清偿拖欠农民工工资1.8亿元。加大了大案、要案查处力度，立案查处各类违纪案件340件。“四五”普法总结验收工作顺利完成。市人防办被授予全国人民防空先进单位称号。民族宗教、外事侨务、对台事务、国家安全、国防教育、民防等工作进一步加强。

　　各位代表，过去的一年，在市委的正确领导下，在市人大、市政协的有力监督和支持下，我们渡过了一个又一个难关，战胜了预想不到的困难，取得了来之不易的成绩，充分显示了阜新人民团结一心，敢于战胜各种困难和挑战的信心和力量。在此，我代表市人民政府，向全市各族人民，向所有参与、关心和支持阜新转型发展的同志们，向在困难之际伸出援助之手的各界朋友们，表示崇高的敬意和衷心的感谢！

　　在充分肯定成绩的同时，我们也清醒地看到，阜新经济社会发展还存在不少问题和困难。经济社会发展中的一些长期性问题和深层次矛盾依然存在，经济结构调整任务艰巨；具有牵动作用的大项目、新项目少，项目储备不足；国企改革难度大，任务重；利用外资规模较小，质量不高；经济增长方式粗放，自主创新能力不强；财政困难局面没有根本改变，收支矛盾更加突出；就业问题和困难群众的生产生活问题还没有得到根本解决，社会稳定压力仍然较大；个别政府工作人员办事效率不高，服务意识不强，政府自身建设有待于进一步加强。对于这些问题，我们必须高度重视，采取更加有力措施，认真加以解决。

　　二、抢抓“十一五”重要战略机遇期，全面推进阜新转型复兴

　　当前，我国经济社会发展正处于一个十分重要的战略机遇期。“十一五”期间，阜新将迎来新的发展机遇，进入新的重要时期。中共中央关于制定第十一个五年规划建议中，将促进资源型城市经济转型作为东北老工业基地振兴的重点之一，阜新经济转型面临新的政策机遇。随着比较优势产业规模的不断扩大，重点骨干企业带动能力的进一步增强，将推动我市主导产业和支柱产业进入新的发展阶段。沿海发达地区及中部产业集聚地区正在进行新一轮的结构调整和总量扩张，将更加有利于我市引进和承接一批产业。省委、省政府提出加快辽西沿海经济区建设，特别是沈彰、阜铁、阜朝等高速公路的建设，将使我市区位优势明显提升。我们要紧紧抓住“十一五”期间面临的发展机遇，在东北老工业基地振兴中，全面推进阜新转型复兴。

　　“十一五”时期，全市经济社会发展总的要求是：高举邓小平理论和“三个代表”重要思想的伟大旗帜，用科学发展观统领经济社会发展全局，紧紧围绕经济转型这条主线，突出推动超常发展与构建和谐阜新两大主题，全面实施工业兴市、强县壮区、改革开放、科教人才强市和可持续发展五大战略，转变经济增长方式，增强自主创新能力，努力打造“绿色精美活力阜新”，基本实现经济转型目标，全面完成经济社会发展的各项任务，为实现全面建设小康社会的宏伟目标打下坚实的基础。

　　“十一五”期间，经济社会发展的主要目标是：

　　——国民经济持续快速发展。到2010年，全市生产总值达到360亿元，年均递增17%。地方财政一般预算收入达到16亿元，年均递增17%以上。全社会固定资产投资累计完成600亿元以上，比“十五”时期增长1.5倍。

　　——经济结构调整取得明显成效。“主导产业+支柱产业”的经济格局基本形成，主导产业占工业增加值的比重达到30%以上；支柱产业占工业增加值的比重达到60%。

　　——经济增长方式进一步转变。科技进步在全市经济增长中的贡献率稳步提升，高新技术产业在全市整体经济中的比重不断提高；单位国内生产总值能源消耗比“十五”期末下降20%左右；循环经济形成若干个完整的产业链条。

　　——新农村建设稳步推进。农村经济结构进一步优化，非农业产值占农村社会总产值的60%以上；“百万农民增收致富工程”主要指标基本完成；力争实现村村通油路和村村通有线电视；农村城镇化水平达到22%以上。

　　——改革开放取得新成果。两年内全部完成国有企业改制；民营经济比重达到70%以上；利用外资额和外贸出口额年均增长25%和20%以上。

　　——城乡市场进一步繁荣。全市社会消费品零售额年均增长18%。

　　——城市面貌明显改观。城市框架扩大到80平方公里；高速公路总里程达到265公里；沉陷区治理和棚户区改造工程全部完成。

　　——生态环境进一步改善。全市森林覆盖率达到40%以上；城市建成区绿化覆盖率达到42%以上；城市空气环境质量二级标准天数达到80%以上。

　　——人民生活水平普遍提高。实现就业再就业15万人以上；城镇居民人均可支配收入达到13000元，农民人均纯收入达到5600元，年均分别增长15%和13%。

　　——社会各项事业全面进步。科技、教育、文化、卫生、人口与计划生育、体育、旅游、新闻出版、广播电视等社会事业蓬勃发展。

　　实施“十一五”规划，是一项系统工程，要站在全局和长远高度，在工作中牢牢把握以下三个要点：

　　第一，坚持把推进经济转型作为主线，促进主导产业和支柱产业优化升级。围绕构筑“主导产业+支柱产业”经济格局，开展“工业年”活动，实施工业“5510”工程。在“十一五”期间，全市要培育壮大50个销售收入超亿元的骨干企业群体，引进建成50个销售收入超亿元的重点工业项目，培育引进10个销售收入10亿元以上的优势产业集群。

　　第二，坚持以项目为核心，建设一批事关阜新长远发展的重大基础设施建设工程和重大项目。加大重点领域、重点产业项目建设力度，通过组织实施一批具有重大牵动作用的工程和项目，夯实加快发展的基础，增强可持续发展能力。要紧紧围绕国家产业布局、政策导向，规划、论证、实施一批对经济社会发展具有推动作用的重大项目。

　　第三，坚持以人为本，统筹经济社会发展，加快建设和谐阜新。要注重各项社会事业发展，为经济发展提供强有力的精神动力和智力支持。要以实现人的全面发展为最终目标，在统筹城乡发展、提高人民生活水平、为人民群众办实事等方面取得明显成效。推进清洁生产，发展循环经济，增强全民节约意识，建设资源节约型和环境友好型城市。

　　三、坚持以经济转型为主线，全面完成今年各项工作任务，实现“十一五”良好开局

　　2006年，是“十一五”规划开局之年，做好全年工作，事关全局，意义重大。今年我市国民经济和社会发展主要预期目标是：全市生产总值增长20%；全社会固定资产投资增长23%；地方财政一般预算收入增长15%以上（可比口径）；规模以上工业增加值增长24%；直接利用外资增长30%，出口创汇增长21%；社会消费品零售总额增长16%；城镇居民人均可支配收入增长15%以上，农民人均纯收入增长15%以上。重点抓好以下十个方面的工作。

　　（一）落实“工业兴市”战略，深入开展“工业年”活动，全力推进工业经济实现新的跨越

　　第一，围绕三个重点，全力推进“工业年”活动。一是加快培育壮大一批骨干企业群体，全市形成销售收入超亿元工业企业30户。阜矿集团重点推进八道壕矿等三个矿井改造，销售收入达到28亿元；阜新发电厂完成三期技改1台机组并网发电，销售收入达到10亿元以上；阜新双汇扩大肉制品生产及生猪屠宰能力，销售收入达到15亿元；阜新伊利、美中鹅业等10户企业销售收入突破2亿元；飞雪糖业有限公司等17户企业销售收入达到亿元以上。二是加快引进建设一批重点工业项目，新引进建设投资3000万元以上项目50个。三是加快培育引进一批重点产业集群，重点培育销售收入超亿元产业集群10个。玻璃、电子、氟化工、装备制造配套、服装、玛瑙等已初步成型的产业集群，要做大做强；同时，结合资源、产业比较优势，引进建设一批产业集群。

　　第二，把工业园区建设作为项目建设和产业发展的重要载体和平台。一是加快两区建设。经济技术开发区要进一步完善基础设施建设，不断扩大引资规模，提高项目质量；国家农业科技园区要争取列入省高新技术产业开发区，在引进高科技项目、产业集群项目上实现新突破。二是推进玻璃、电子、氟化工、机械加工、橡胶等工业园区建设，加快项目进区进度。开工建设日熔化420吨一窑三线超白玻璃生产线等一批项目，抓好环宇公司年产150万平方米PVG高强力输送带等一批项目竣工投产。三是规划论证建设一批新的工业园区，积极引导相关企业向园区集聚。

　　第三，建立一套“工业年”组织责任体系。一是实行市级领导包重点项目、重点部门包重点产业制度，形成市领导和重点部门承包产业的工作格局。二是完善政策扶持机制，制定鼓励政策，对重点产业、重点企业、重点项目建设给予支持。三是实行分工负责，市经委等部门要各司其职、通力协作。四是全方位、多渠道争取资金，大力引进外资，搞好融资。同时,要采取有效措施，挖掘民间资本，激活和吸引民间资金。五是加大固定资产投资力度，把固定资产投资作为重要考核指标，纳入“工业年”考核体系之中。

　　（二）围绕建设社会主义新农村，加快农业结构调整，推进农业生产再上新台阶

　　第一，以三个一批为重点，进一步调整优化农业结构。一是以阜新双汇等骨干农产品加工企业为依托，壮大瘦肉型猪、奶业、肉鸡等一批产业；二是以现有和在建的农产品加工企业为依托，推进肉羊、肉驴等一批产业；三是以引进和建设一批农产品加工企业为依托，培育蛋鸡饲养、林产品加工、饲料加工等一批产业。全市猪、牛、羊、禽饲养量分别达到350万头、26万头、160万只、4000万只，肉、蛋、奶产量分别达到32万吨、8万吨、8万吨。新建“四位一体”生态模式户3000户。新植果树1万亩。种植牧草5万亩。全地区农产品基地基本实现无公害化，新增绿色食品认证8个。

　　第二，围绕农业结构调整、农产品基地建设、特色产业开发，以户均一个致富项目为载体，全面推进百万农民增收致富工程。年内新增从事致富项目的户数5万户。加大劳务输出工作力度，全年农村劳务输出18万人次。

　　第三，强化农村社会化服务体系。重点抓好动物疫病防控长效机制建设，形成市、县、乡、村四级防控体系。继续完善良种繁育、绿色食品检测、技术服务推广体系建设。新建动物改良站（点）150个。鼓励和引导农民发展各类专业合作经济组织，创办农村专业合作组织协会20个。抓好“五大示范工程”，农业标准化生产推广面积达到2万亩；有机食品示范点要增加作物品种，面积达到300亩；推广林下经济开发成功模式12个，面积达到100万亩；“四位一体”生态模式户及节水示范工程要适当扩大规模。继续推进农业园区建设。

　　第四，加强生态建设和农业基础设施建设。完成植树造林和封山育林70万亩，其中“三北”造林10万亩，退耕还林40万亩。实施荒山荒沙封育促进工程20万亩。继续推进封山禁牧工作。实施农村饮水安全工程，抓好防氟改水，解决农村6.33万人饮水问题。新增灌溉有效面积5万亩。完成水土流失治理面积25万亩。完成机械化保护性耕作面积70万亩。

　　第五，加强村容村貌整治和环境建设。进一步完善乡镇、村屯规划，重点抓好乡镇驻地和村屯绿化、美化，加强村屯公路等基础设施建设。抓好试点，搞好示范，推进社会主义新农村建设。

　　（三）积极扩大对外开放，在招商引资政策环境、规模质量上实现新突破

　　第一，改进招商引资方式。突出小分队招商，全市各级都要组建专业招商小分队，有针对性地到国内外企业、集团开展“敲门招商”、“专业招商”；突出高层走访招商，各级领导干部都要有选择、有重点地出国、出境、出市，走访境内外大企业、大集团；突出拉网式招商，每个产业集群重点联系2000户企业，其中，通过网上联系1000户，上门联系1000户。

　　第二，突出生产型项目。围绕我市优势产业的前延后伸和相关配套产业，重点做好提升产业、产品结构项目和资源开发利用项目等生产型项目的招商，促进产业优化和升级。要突出“十一五”期间我市重点培育的10个优势产业集群，包装、推介和引进一批项目和企业。要把园区招商作为招商引资工作的重中之重，尽快形成园区集聚效应。要进一步提高招商引资项目质量，调整全市对外开放有关奖励办法，重点鼓励生产型项目和产业集群项目的引进。

　　第三，突出重点国家和地区。把利用外资与推进我市企业与境内外大企业、大集团合资合作结合起来，充分利用已经建立起来的各种渠道和网络，积极组织参加境外地区的招商活动。紧紧盯住长三角、珠三角等国内发达地区，河北、山东、河南等产业集聚地区，积极承接和引进一批产业转移、产业辐射项目。同时，充分发挥与辽西各市及辽宁中部城市群在产业、资源、区位上的互补性，积极推进区域经济合作。

　　第四，调整出口产品结构，拓宽外贸出口渠道。对飞雪木业、振隆土特产、北方毛革等重点农产品加工企业给予扶持，促进出口企业和出口产品基地规模扩大。在继续巩固香港、日本、韩国等市场的同时，针对我市出口产品市场需求，积极拓宽新的国际市场。认真贯彻“走出去”战略，鼓励有实力、有需求的企业发展对外经济技术合作。

　　（四）围绕加快体制机制创新，积极推进各项改革，大力发展民营经济

　　加快国有企业改革进程。全面完成地方国有工业企业改制，基本完成商贸、物资、供销、交通、建筑企业改制，粮食企业的改制要完成80%以上，公益性、特殊行业的企业改革要取得突破性进展。全面启动已列入国家正式破产计划的市电缆厂、市冶金备件厂等5个破产项目。抓好海州矿破产收尾工作，加快重组进程。深化农村各项改革，稳步推进农村税费配套改革，完善土地流转办法，加快林权制度改革，做好小型水利产权制度改革后的完善工作。按照省统一要求，继续做好事业单位的分类改革，推进具有行政职能的事业单位改革。

　　大力推进民营经济加快发展。按照培育优势企业、壮大骨干企业、发展中小型企业的要求，突出规模企业和纳税大户的培植，积极引导和鼓励民营企业增加投入，参与国企改制重组，不断扩大规模，增强实力。坚持“软环境争优，硬环境创优”，制定出台支持民营经济发展的具体措施，进一步优化民营经济发展环境。全年新增销售收入超5000万元的企业10户，其中超亿元的3户。

　　（五）全面落实支持县区经济发展各项政策，促进县区经济做大做强

　　坚持以工业化为主导，实施“强县壮区”战略，落实支持县区经济发展的各项政策，加快发展县区经济。一是要立足现有优势，培育壮大支柱产业和骨干企业。要大力发展农产品加工业、劳动密集型工业和特色优势工业，构建具有区域特色的支柱产业，每个县区发展壮大2个销售收入超亿元的骨干企业。二是发挥资源优势和产业比较优势，培育发展产业群体，每个县区培育引进1-2个产业集群。三是按照“高起点规划、高标准建设、高效能管理”的原则，积极发展园区工业。各县区要围绕培育产业集群，规划建设1-2个工业园区。四是加快发展乡镇、街道经济。通过加快项目建设，开拓税源，增强乡镇、街道经济实力。具备一定基础的乡镇都要进入税收百万元乡镇行列，同时要培育10个财政收入超500万元乡镇。

　　（六）加快构筑具有阜新特色的现代服务业，增创经济发展新优势

　　抓住国有商业企业改制机遇，加强与知名商贸流通企业合资合作，重点推进百货大楼、新世纪商城等对外合作招商。启动东市场商业区改造，加快推进新天地商业广场和时代广场建设。开工建设市蔬菜批发市场、绿色农产品批发市场一期、国家农业科技园区花卉市场等一批专业市场，对市果品市场进行改扩建。加快推进“万村千乡”市场建设工程，年内新增各级连锁超市200家。加快发展以社区服务业为重点的新兴服务业，创办社区便民利民服务企业100家。稳步发展房地产业，年内开发180万平方米、竣工80万平方米（含棚户区）。

　　完成《阜新市旅游发展总体规划》编制工作。做好海棠山、瑞应寺、大清沟重点景区申报国家AAAA级风景区工作。全面启动创建“中国优秀旅游城市”活动，完成阶段性目标。进一步加快矿区特色旅游开发进程。

　　（七）认真做好财税金融工作，为推进经济转型提供资金支撑

　　积极扶持财源项目，培育新的税收增长点，巩固基本财源。制定出台具体办法，支持税收贡献大的企业和项目。进一步整合专项资金、非经营性资产和资源性资产等财力资源，集中财力，支持重点项目。要加强财税管理，规范税收秩序。各级税务部门要加大税收征管力度，推进依法治税，做到应收尽收。各级财政部门要积极组织非税收入，大力挖掘增收潜力。优化支出结构，坚决压缩不合理开支，确保重点支出。要冻结人事编制，严格控制党政机关购买小汽车，坚决控制会议费、办公费等一般性财政支出，真正树立起过紧日子的思想。加强对财政性资金使用情况的监督检查，严格审计监督，用好管好各项资金。要切实加强金融监管，维护金融业稳健运行。加强银企合作，加大对重点项目的信贷支持力度，继续做好银行不良贷款核销工作，为地方经济建设提供强有力的金融支持。农村信用社要围绕农业结构调整，继续做好小额贷款发放工作。

　　（八）推进基础设施建设，加强城市环境综合整治，进一步提升阜新城市形象

　　围绕城市建设管理上台阶、上水平、上档次的目标，全面开展“城市建设提高年”活动。全年重点推进五大工程建设。一是交通网络建设工程。开工建设城市大外环，进一步拓展城市发展空间。沈彰、阜铁、阜朝三条高速公路阜新段路基和中小桥涵工程完工，力争启动彰武至通辽高速公路工程。巴新铁路重点控制工程开工。阿金公铁立交桥8月1日竣工通车。完成村通油路120公里。二是推进棚户区改造工程。全年拆迁101万平方米，全面完成棚户区改造拆迁任务，新建住宅160万平方米，竣工60万平方米。抓好采煤沉陷区一期工程收尾工作和各项配套工程建设，10月底工程全部竣工。三是城市管网改造工程。继续加大对涉及群众切身利益的“水、气、热”三大管网改造力度。四是城乡环境改善工程。结合矿区棚户区改造，加强市区南部基础设施建设。引白水源工程全面开工建设。细河治理二期工程开工，五号坝竣工蓄水。污水处理厂正式运行。继续抓好街巷路改造和亮化。全面启动海州露天矿综合整治工程，完成新邱东、西排土场土地复垦工程，开工建设采煤沉陷区治理复垦项目。重点抓好大外环、采煤沉陷区和城市规划区绿化，完善城市街巷路和重点绿地绿化，城市植树35万株，新增公共绿地面积100万平方米。五是城市环境整治工程。以强化规划管理、交通整治、街巷卫生、车辆停放为重点，进行全方位的城市环境整治和管理。通过市场化运作，建设和改造一批公厕。进一步改善城区环境空气质量，确保全年2/3天数达到国家二级环境空气标准。

　　（九）加强社会主义精神文明建设，积极发展科技教育等各项社会事业

　　广泛开展群众性精神文明创建活动，加强社会公德、职业道德和家庭美德教育，努力提高人民群众道德观念、法律意识和文明素质。大力开展文明社区建设，组织“科技、法律、文化、卫生、体育”进社区活动。组织开展市民“文明行为”规范教育，引导市民摒弃各种不文明行为，养成良好的道德习惯，推动良好社会风气的形成。

　　巩固科技共建成果，积极引进推广新技术、新项目，增强自主创新能力。落实省内院校合作、海外学子创业、省民间资本对接等科技项目。完善以农业科技成果转化中心为核心的农业科技研发网络，加速科技成果转化。加快技术创新，完成高新技术产业产值18亿元，增长20％。积极推进新一轮普及九年义务教育工作，加快职业教育改革和发展步伐。巩固普通高中招生规模，继续抓好中、高等学校招生工作。搞好民族教育基地建设。支持和规范民办教育。加强农村初中控辍保学工作。进一步做好城乡校舍维修和改造工作。抓好多层次人才队伍建设。落实“一村一名大学生”人才培养计划。启动城乡初级人才培训工程。实施聘请国外专家项目15项，完善市级农业引智成果推广体系。进一步完善吸引人才、留住人才的政策措施。

　　积极做好阜新玛瑙手工技艺国家级非物质文化遗产申报工作。进一步完善现代京剧《血胆玛瑙》，认真做好参加中国京剧节和中国艺术节各项准备。完成“有线电视村村通”6万户，发展数字电视用户1万户。完成市中医院病房楼、市卫校综合教学楼建设工程。抓好农村公共卫生、基本医疗服务体系规划建设工作。推进阜蒙县新型农村合作医疗试点扩面，启动彰武县新型农村合作医疗试点。继续抓好篮球、赛艇、举重三个国家级体育后备人才基地建设，广泛开展全民健身活动。

　　加强人口和计划生育工作，稳定低生育水平。积极开展创建节约型机关、节约型企业、节约型社区等活动，努力建设节约型社会。关心和支持妇女、儿童、老年人、残疾人等事业，启动全市第二轮修志工作，继续做好统计、气象预报、防震减灾、文物保护等工作。

　　（十）认真解决人民群众生产生活问题，推进和谐阜新建设

　　继续坚持就业优先战略，重点推进棚户区下岗失业人员、“零就业家庭”、采煤沉陷区搬迁的下岗失业人员、并轨失业人员、“4050”人员实现再就业。全年培训下岗失业人员2万人，实现实名制就业5万人。不断完善社会保障体系，抓好保险扩面工作。基本养老保险扩面要完成市级统筹任务，扩大医疗保险覆盖面，加大失业保险基金的筹集力度。建立分类救助、定期定额救助、重大疾病医疗救助、低保检查制度等低保配套制度，确保农村最低生活保障制度和城市重大疾病救助制度顺利实施。70%社区卫生服务站达到省示范单位标准。对60个村实施开发式扶贫，移民扶贫300户，实现10万人脱贫。进一步完善慈善总会功能。认真解决城乡贫困家庭子女就学问题。强化住房公积金归集，加大政策性低息贷款力度，满足中低收入家庭购房需求。

　　进一步加强民主法制建设。自觉接受人民代表大会及其常委会的依法监督和人民政协的民主监督，认真办理人大代表议案、建议和政协提案。加强与民主党派、工商联、无党派人士和人民团体的联系，重视并听取其意见和建议。充分发挥工会、共青团、妇联和科协等组织在安置就业、扶贫济困和科技进步等方面的积极作用。巩固“四五”普法成果，提高全民法律素质。整顿和规范市场经济秩序，重点开展食品卫生监督检查。认真落实民族区域自治法，加大支持力度，促进民族地区经济社会健康发展。继续做好外事、侨务、对台、人防、民防工作。巩固和发展军政军民团结。深入开展平安县、平安区活动，全面加强社会治安防控体系建设。完善信访工作制度和机制，落实信访包案制度。严格落实安全生产责任制和责任追究制，坚决防止重特大事故的发生。

　　四、努力建设依法行政、高效务实、亲民诚信、阳光透明、清正廉洁的政府

　　按照市委关于加强软环境建设的要求和市人大的有关建议，坚持从制度建设入手，推进政府自身建设。

　　第一，建设依法行政政府。全面落实省政府取消、调整第三批审批项目和取消第一批年检项目的决定，取消一批非行政审批项目。对取消年检的项目加强日常监管，防止管理脱节。全面推行重点单位软环境评议制和行政执法责任制，重点加强对行政“一把手”的考核和责任追究。进一步做好行政复议应诉工作，切实保护广大公民、法人和其它组织的合法权益。不断规范行政执法行为，切实解决多头执法问题，严肃查处各种违法违纪行为。

　　第二，建设高效务实政府。全面开展以办事效率、服务质量和工作纪律为主要内容的执法监察、行政效能监察。继续强化市行政服务中心功能，加快非行政许可审批项目进厅步伐。加快各县区行政服务中心建设，与市行政服务中心实现联网，实现全市行政服务工作统一监管。完善企业投诉中心建设，加大企业投诉中心受理、查处案件力度，切实维护企业合法权益。进一步发展和规范各类中介组织和机构，对依附于行政机关的各类中介组织和机构一律剥离。加强应急体系建设，切实提高政府有力保障公共安全和迅速处置突发公共事件的能力。

　　第三，建设亲民诚信政府。从解决最直接、最具体的问题入手，扎实做好人民群众关心的实事。市、县政府要确定一系列为民办的实事，年初公布事项，年终通报结果。继续落实好市长接听公开电话、直接接待上访等制度，定期公布办理结果。加快建立和完善重大问题集体决策、专家咨询、社会公示、听证等制度，研究建立决策后评估机制，不断提高决策的科学化、民主化水平。加强信用体系建设。行政机关所作的公开承诺，必须全部兑现；违背承诺的，要按照相关规定追究有关责任人的具体责任。强化市民投诉中心网络建设，树立市民投诉中心权威。继续办好《行风热线》。在阜新电视台开办《民声直通车》栏目。

　　第四，建设阳光透明政府。进一步完善新闻发布会制度和政务信息公开制度，力求全面、及时、准确，增强政府行为透明度。政府部门所有的政策、办事程序、审批事项都要通过政府网站、政府公报、新闻媒体等多种方式及时向社会公开。加强政府信息平台建设，加强政务公开电子大屏幕的管理，“阜新新闻网”要及时提供全部政策信息服务，促进信息资源共享。进一步畅通听取群众意见的渠道，形成群众意见和建议的办结、反馈、回复制度。

　　第五，建设清正廉洁政府。建立健全教育、制度、监督并重的惩治和预防腐败体系，加大从源头上预防和治理腐败的力度。深入开展专项治理，切实纠正部门和行业不正之风。强化政府系统内部的层级监督，支持监察、审计部门依法独立履行监督职责，围绕政府重点工作和群众关心的热点问题，开展执法监察和审计监督。加大对政府投资项目、领导干部经济责任审计力度，大力查处违法违纪案件。深入开展“为民、务实、清廉”主题教育活动。强化领导干部廉洁自律意识，规范行政行为。

　　各位代表，阜新的未来发展机遇与挑战并存。要在新的起点上实现更快更好的发展，必须付出艰苦的努力。我们要在中共阜新市委的领导下，高举邓小平理论和“三个代表”重要思想伟大旗帜，以更足的干劲、更实的作风，带领全市人民在阜新转型复兴的伟大征程上迈出新的步伐！
